# Endometrial cytokine expression from clinically suspected genital tuberculosis patients at tertiary care hospitals in Dhaka

**DOI:** 10.1016/j.jctube.2022.100301

**Published:** 2022-01-31

**Authors:** Sheikh Joly Ferdous Ara, Sharmeen Ahmed, Ahmed Abu Saleh, Md. Maruf Ahmed Molla, Sharmin Chowdhury, Shaheda Anwar

**Affiliations:** a Z.H. Sikder Women Medical College & Hospital, Dhaka, Bangladesh; b Microbiology & Immunology Department, Bangabandhu Sheikh Mujib Medical University, Shahbag, Dhaka, Bangladesh; c National Institute of Laboratory Medicine and Referral Center, Dhaka, Bangladesh; d Infection Control Officer, United Hospital, Dhaka, Bangladesh

**Keywords:** FGTB, Z-N staining, L-J culture, IFN-γ, TNF-α, ELISA

## Abstract

**Objective:**

The objective of this study was to measure gamma interferon (IFN-γ) and tumor necrosis factor alpha (TNF-α) expression in endometrial tissue and/or aspirate from suspected genital tuberculosis patients with ectopic pregnancy and infertility in Bangladesh.

**Methodology:**

A total 78 women of clinically suspected genital tuberculosis patients were enrolled as study population. These patients underwent manual vaccum aspiration (MVA) procedure, and endometrial tissues and/or aspirates were collected. Ziehl -Neelsen staining (Z-N staining) and Lowen–Stein Jensen (L-J) culture were done to detect *Mycobacterium*. The study participants were categorized as genital tuberculosis positive cases, genital tuberculosis negative cases and presumptive for tuberculosis cases based on the case definition used in this study. TNF-α and IFN-γ were measured by ELISA. Statistical analysis was done using SPSS (version-22).

**Results:**

Out of 78 participants, pro-inflammatory cytokines IFN-γ and TNF-α were significantly increased in TB positive patients than TB negative patients (p < 0.05). IFN-γ value of TB positive patients (41.26 ± 41.05) was higher than TB negative (22.94 ± 44.51) patients. TNF-α value (44.31 ± 64.22) of TB positive patients was higher than TB negative (15.86 ± 41.45) patients. IFN-γ and TNF-α value of presumptive for tuberculosis cases were not statistically significant. According to ROC analysis, cut off value for IFN-γ was 23.5 and for TNF-α was 10 with highest sensitivity and specificity of 66.7%, 89.3%, and 66.7% and 73.1% respectively.

**Conclusion:**

IFN-γ and TNF-α were significantly higher in TB positive patients and it may act as a potential biomarker for diagnosis of genital tuberculosis.

## 1 Introduction

Pulmonary tuberculosis (PTB) remains the commonest and the most infectious type of tuberculosis (TB), but extra-pulmonary TB (EPTB) is becoming more prevalent, especially in women of reproductive age group throughout the world [1]. The prevalence of EPTB in Bangladesh is about 11% [2]. According to 2016 statistics, EPTB has increased rapidly ranging from 8% in the WHO Western Pacific Region to 24% in the WHO Eastern Mediterranean Region [3]. EPTB can potentially affect any organ of the body but there are particular sites where it can localize more frequently like genitourinary (GU) system [4]. Females with genitourinary tuberculosis comprise approximately 0·5% cases of all TB cases and among genitourinary cases 50 % are genital TB cases [5]. FGTB remains undetected for a long time because of its silent nature and lack of confirmatory diagnostic parameter [6].

Most women are diagnosed as genitourinary tuberculosis (GUTB) during investigations for primary or secondary infertility. Several studies were carried out in order to compare the different methods such as Mantoux test (MT), hysterosalphingography (HSG), laparoscopy, ultrasonogram (USG) [7], histopathological examination (HPE), detection of acid-fast bacilli (AFB) in smear, Lowenstein-Jensen (LJ) culture, BACTEC culture (Bactenecin), polymerase chain reaction (PCR) in diagnosing genital tuberculosis [8]. FGTB is a pauci bacillary disease and diagnostic dilemma arises due to varied clinical presentation and lack of definitive diagnostic procedures. None of the diagnostic techniques available for the detection of female genital tuberculosis (FGTB) are 100% accurate. Conventional methods have slow and low detection rates. Sampling sites may not represent the infected area and the infected site can be missed due to sparse number of *Mycobacteria*. PCR results may either false positive or false negative as it cannot detect dead or live bacilli in the specimen [9], [10].

In All India Institute of Medical Sciences (AIIMS), New Delhi reported that 26 % women of infertility suffered from FGTB and incidence of infertility in FGTB was 42.5% [11]. In Pakistan, the frequency of GTB was 2–10% [12], [13] and 42.5% patients with infertility suffered from GTB [14]. Some authors reported 13.2% and 35.29% ectopic pregnancy as a cause of genital tuberculosis respectively [15], [16]. In a clinical study, 34% menstrual disturbances among genital tuberculosis (GTB) patients have been reported [17].

Fallopian tubes are the likely source of initial infection and both tubes are involved in 85–100% of cases [7], [8]. The uterus is involved in 50–70% of cases predominantly affecting the endometrium and occasionally the myometrium [17] .The reproductive outcome in patients of tuberculosis has been described as poor and the results vary from 16 to 38.2% [8], [18]. Among affected individuals active tuberculosis arises only in 10% of cases and generally a clinically asymptomatic latent persistent infection develops [19].

Release of inflammatory cytokines such as interleukin (IL-2), interferon gamma (IFN-γ), and tumor necrosis factor alpha (TNF-α) may lead to implantation failure or recurrent miscarriage, if the host tissue fails to resist this trauma [6]. Some observational studies [19] and case reports [20] indicate that any intervention carried out to achieve a pregnancy can activate a latent tubercular infection and lead to implantation failure. *M. tuberculosis* specific T cells secrets IFN-γ and which have been found more frequent in individuals with active tuberculosis [21], [22]. IFN-γ causes activation of macrophages in vascular endothelium and promotes plasma extravasations [23]. Low IFN-γ production may impair anti mycobacterial response against FGTB infection which renders individuals more susceptible to tubercular bacillus infection other than pulmonary TB [24]. In extra pulmonary TB, tumor necrosis alpha (TNF-α) possess a more pro-inflammatory cytokine profile compared to pulmonary TB [25]. MTB infestation in endometrium excites an inflammatory process. It may be mild but induces Th1 cytokines. It is well known that for successful implantation of blastocyst, Th2 bias is specifically needed. Inflammatory environment in the endometrium may inhibit down-regulation of Th1 bias and up-regulation of Th2 bias. This leads to increased Th1 cell cytokines in endometrium, making it non receptive to embryo and causes recurrent implantation failure [6]. IFN- γ and TNF-α may become an indicator of local presence of MTB and their harmful influences in the reproductive function of suspected genital tuberculosis patients [26]. MTB infection causes mycobacterium colonization in endometrial or tubal wall surface and reproductive failure. The findings of increased prevalence of IFN-γ and TNF-α in endometrial tissue and aspirate in TB positive cases may provide a possibility to become an important clinical indicator of endometrial hostility [26], [6]. But there is no satisfactory data regarding cytokine expression among FGTB patients in Bangladesh.

The aim of this study was to diagnose genital TB patients by Lowenstein-Jensen culture and Ziehl - Neelsen staining and observe endometrial expression of IFN-γ and TNF-α among them by ELISA.

## 2 Methodology

### 2.1 Study design and sample selection

This was a cross sectional study. A total of 69 patients with ruptured pregnancy, attending Department of Obstetrics and Gynaecology, Dhaka Medical College & Hospital and 9 infertility patients of Department of Reproductive Endocrinology & Infertility, BSMMU (for a total of 78 participants), who were suspected, upon taking a detailed history and performing relevant investigations, as genital tuberculosis cases by clinicians, were enrolled in this study. Since women suffering from genital tract tuberculosis might present with symptoms similar to PID, researchers adopted a mixed approach including taking detailed history, looking for evidence of bacterial infections, and performing routine blood, urine tests and ultrasound scanning to presumptively differentiate between PID and FGTB. Population enrolled in this study were in the reproductive age group ranging from 22 to 40 years.

### 2.2 Ethical consideration

The objectives of the study, procedure, risks, benefits, and privacy issues were explained to the patients. A written informed consent was taken from the participants. All data were masked and stored in a password protected laptop. The study was ethically approved by Institutional Review Board at BSMMU.

### 2.3 Data and laboratory sample collection

The participants were interviewed individually and all relevant clinical history (patient clinical history, specific data related to risk factors for tubercular infection, family history of tuberculosis infection, clinical history and treatment history about ectopic pregnancy, infertility, menstrual disturbances were documented in a predesigned data sheet. Endometrial tissue was collected from each participant for cytokine analysis. Results obtained from the laboratory methods were also recorded in the data collection sheet.

### 2.4 Case definition

FGTB suspected case was considered as genital tuberculosis positive case by positive L-J culture, considered to be gold standard for TB diagnosis, and Z-N stain. Both L-J culture and Z-N stain negative cases were considered as Non-FGTB cases. Finally, only Z-N stain positive cases were considered as presumptive of TB cases.

### 2.5 Statistical analysis

All the data were rechecked, coded, entered in a data base, and analyzed using SPSS software (Version-22). The categorical variables were expressed as numbers (n) and percentages (%), while continuous variables were expressed as Mean ± Standard Deviation. To observe the association of tuberculosis positive and negative patients with cytokine level (IFNγ, TNFα) Mann–Whitney test was performed. The average absorbance values for each standards and samples were calculated. A linear standard curve was generated by plotting the average absorbance of each standard on the vertical axis versus the corresponding cytokine standard concentration on the horizontal axis. The amount of cytokine in each sample was determined by extrapolating optical density (OD) values against cytokine standard concentrations using the standard curve. For all statistical analysis p-value < 0.05 was considered statistically significant.

## 3 Results

From a total of 78 samples, four samples were excluded from the final analysis because the range of cytokines could not be estimated. Remaining 74 samples were analyzed for IFNγ and TNFα using ELISA. No sample was found to be positive by PCR.

A significant portion of participants (41%) belonged to the age group of 22–26 years. Their mean age was 29 years (Table 1). Out of 78 patients, 21 (26.88%) cases were positive by Z-N staining, L-J or both. About 12 (15.38%) patients were positive for AFB smear microscopy and 4(5.1%) were culture positive only. There were 5 (6.4%) cases positive by both microscopy and culture (Table 2). Out of 78 samples 44 (56.4%) study participants resided in urban areas and 34 (43.6%) resided in rural area.

**Table 1:** Distribution of study population according to age (n = 78).

**Age (in years)**	**Frequency (n)**	**Percentage (%)**
22–26	32	41.0
27–31	20	25.6
32–36	19	24.4
37–40	7	9.0
Total	78	100

**Table 2:** Positivity of different tests for AFB among 78 patients.

**Test positive**	**Number of cases (n)**	**Percentage (%)**
Only Z-N staining	12	15.38
Only culture	4	5.1
Z-N staining and culture both	5	6.4
Total	21	26.88

**Note**: 4 cases were only culture positive and 5 cases were both Z-N stain and culture positive. Total GTB cases were = 9

Based on case definition of TB cases used in the study, among 78 suspected genital tuberculosis cases, nine were found to be tuberculosis positive and 57 cases were tuberculosis negative and 12 cases were presumptive for tuberculosis (Table 3).

**Table 3:** Category of study population according to case definition (n = 78).

Study population	Number of patients (n)	Percentage (%)
TB positive patients	9	11.5%
TB negative patients	57	73.07%
Presumptive for TB	12	15.38%

Level of IFN γ and TNFα were tabulated and comparisons were done between TB positive, TB negative and presumptive of TB patients (Table 4, Table 5).

**Table 4:** Level of IFN γ among study patients.

**Study population**	**IFN γ (pg/ml)**			**P value**
	**Mean**	**Standard deviation (SD)**	**Range**	
TB positive cases (n = 9)	41.26	41.05	0.02–119.3	*0.01
TB negative cases (n = 57)	22.94	44.51	1.35–294.80	
Presumptive for TB (n = 12)	37.99	83.09	2.18–298.3	**0.5

**Note**: IFN γ level was significantly higher among TB positive cases than TB negative cases (*p = 0.01).

IFN γ level was not significantly higher among presumptive for TB cases than TB negative cases (**p value = 0.5).

**Table 5:** Level of TNF-α among study patients.

**Study population**	**TNFα (pg/ml)**			**P value**
	**Mean**	**Standard deviation (SD)**	**Range**	
TB positive cases (n = 9)	44.31	64.22	4.64–202.2	*0.01
TB negative cases(n = 57)	15.86	41.45	0.0–296.50	
Presumptive for TB (n = 12)	15.74	9.96	1.60–26.9	**0.05

**Note:** TNFα level was significantly higher among TB positive cases than TB negative cases (*p = 0.01).

TNFα level was not significantly higher among presumptive for TB cases than TB negative cases (**p value = 0.05).

IFN γ and TNFα level were significantly higher in TB positive patients compared to TB negative and presumptive for TB patients (p value < 0.05) (Table 4, Table 5).

**Fig. 1:**
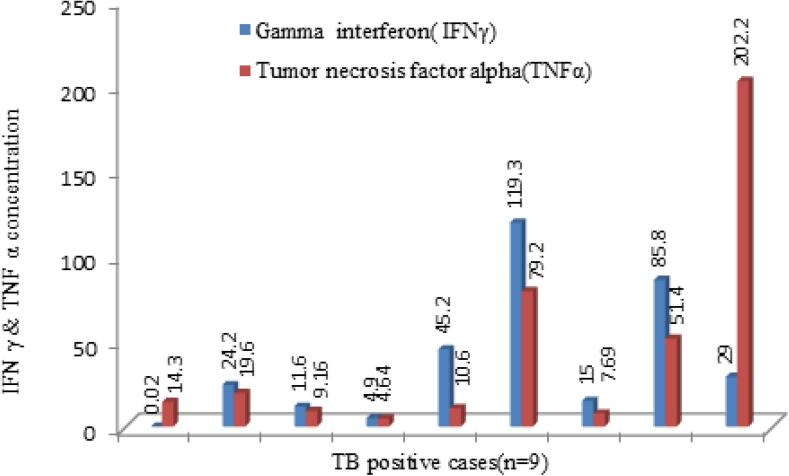
Level of IFNγ and TNFα among TB positive patients (n = 9).

**Fig. 2:**
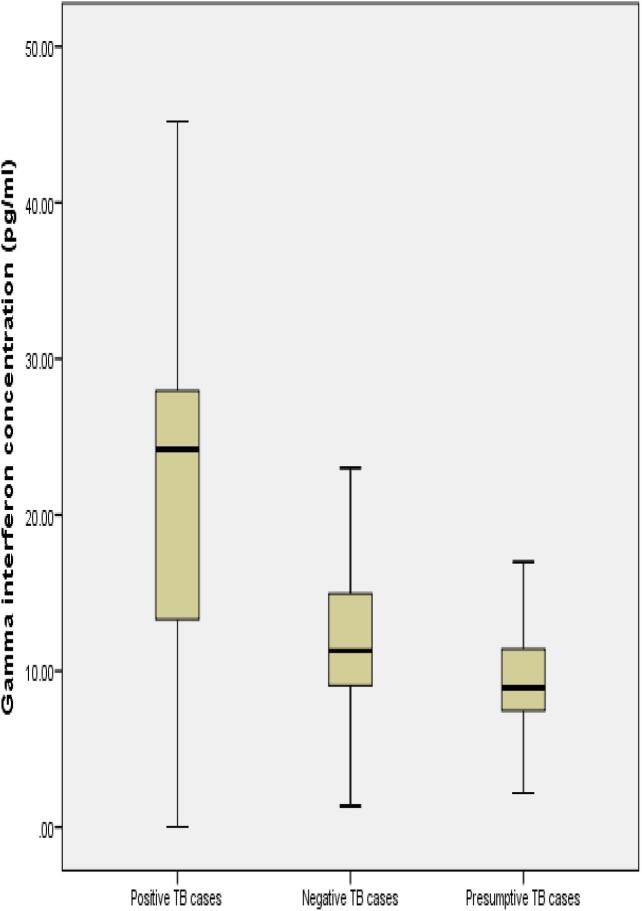
IFN γ median value (23.5 pg/ml) demarked by dark horizontal mark in TB positive cases.

**Fig. 3:**
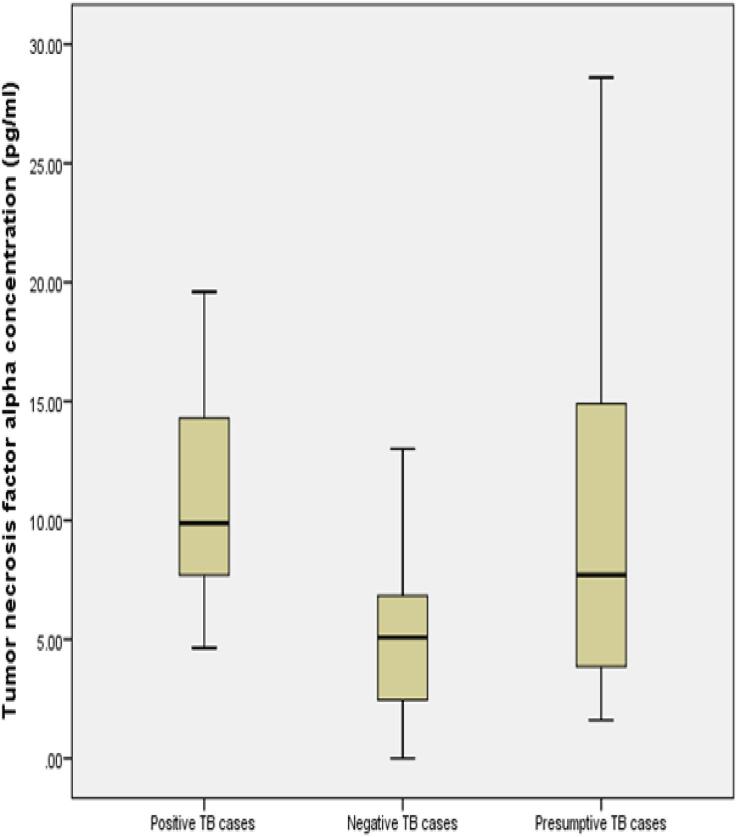
TNF-α median value (10 pg/ml) demarked by dark horizontal mark in TB positive cases.

Fig. 1. shows the level of IFNγ and TNFα in TB-positive cases. The median concentration of IFNγ and TNFα which was demarked by dark horizontal bar (-) were illustrated using a whisker box plot (Fig. 2, Fig. 3). The median value of IFN-γ was 23.5 pg/ml and TNF-α was10 pg/ml among TB positive patients.

**Fig. 4:**
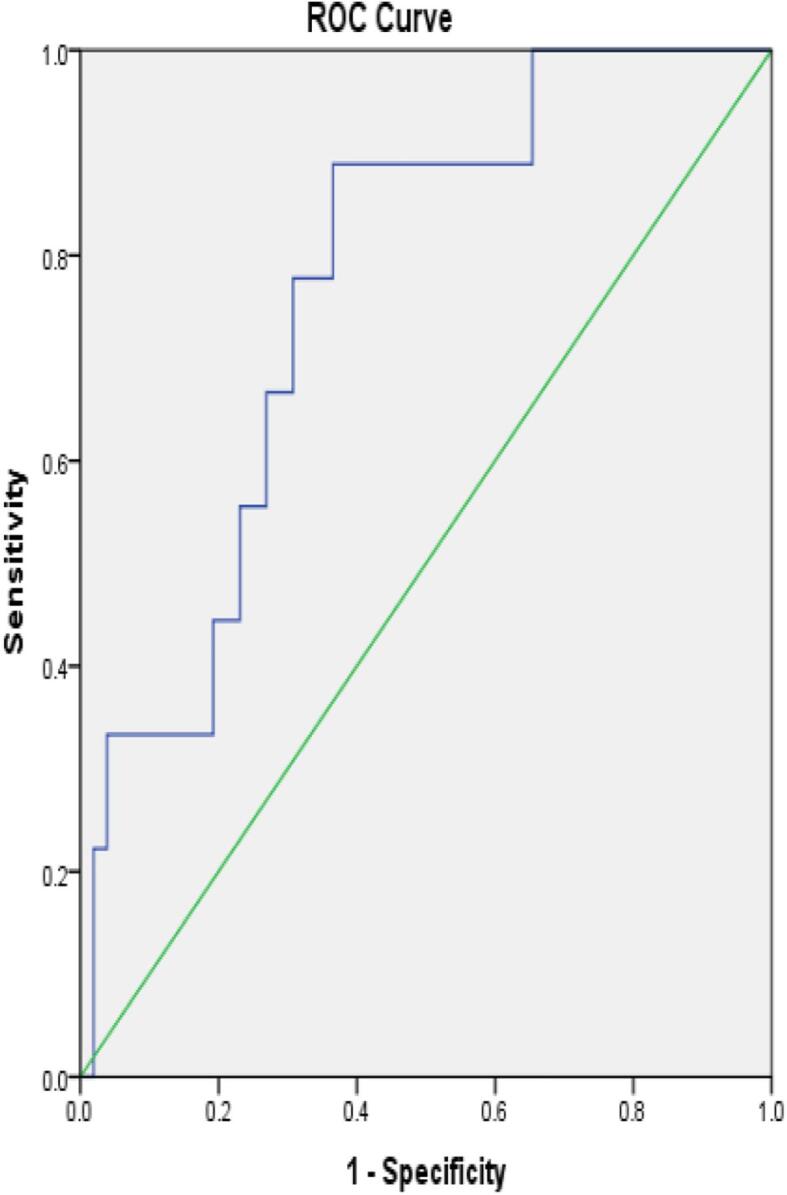
ROC analysis for TNF α among TB positive cases.

**Fig 5:**
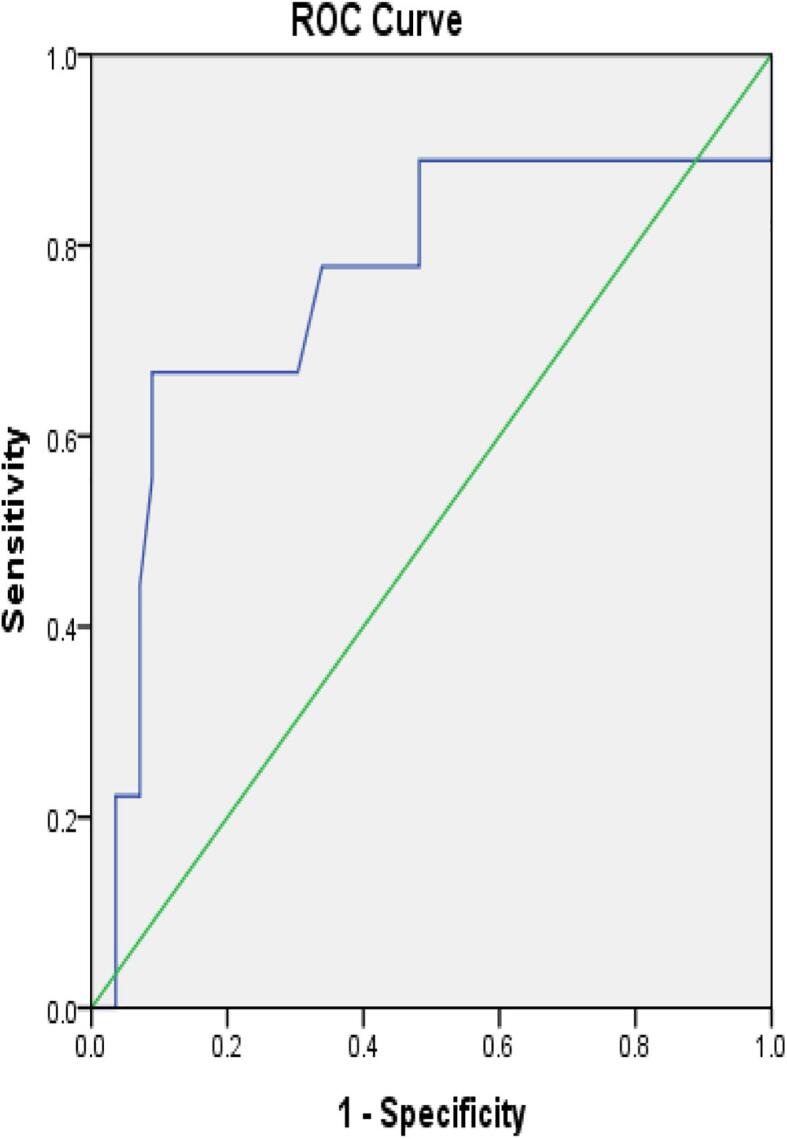
ROC analysis for IFN γ among TB positive cases.

ROC analysis was performed to evaluate diagnostic potential of TNFα and IFN γ (Fig. 4, Fig 5). Among TB positive cases, TNFα level achieved an area under curve (AUC) of 0.767 (95% CI: 0.619–0.915) and IFN γ achieved AUC of 0.757 (95% CI: 0.550–0.964). The TNF-α cut off value was 10 pg/ml with a sensitivity of 66.7%, and specificity of 73.1%. IFN γ cut of value was 23.5 pg/ml with sensitivity of 66.7% and specificity of 89.3% among TB positive cases (Table 6).

**Table 6:** ROC analysis for IFN γ and TNF- α in discriminating TB positive and TB negative patients.

	**Cytokine**	**AUC(95% CI)**	**Cut-off (pg/ml)**	**Sensitivity(%)**	**Specificity(%)**	**P value**
TB positive (n = 9) and	TNFα	0.767(0.619–0.915)	10	66.7%	73.1%%	0.01
TB negative patients (n = 57)	IFN γ	0.757(0.550–0.964)	23.5	66.7%	89.3%	0.01

AUC = Area under curve

Table 7 shows the frequency and pattern of sign-symptoms among TB positive and TB negative patients. About 7 (77.7%) participants had no sign symptoms of genital tuberculosis. 11.1% patients had complaints of oligomenorrhoea and whitish discharge per vagina.

**Table 7:** Sign symptoms of TB cases and non-TB cases.

**Sign & symptoms**	**TB cases (n = 9)**	**Non TB cases (n = 57)**	**Total**
Oligo-menorrhoea	1(11.11%)	19(33.33%)	20(30.3%)
Menorrhagia	0	5(8.77%)	5(7.57%)
Whitish discharge per vagina	1(11.11%)	19(33.33%)	20(30.30%)
History of previous TB	0	3(5.26%)	3(4.54%)
Asymptomatic	7(77.77%)	11(19.29%)	18(27.27%)

## 4 Discussion

Worldwide 10–85% females presenting with infertility are suffering from genital tuberculosis and different sequelae such as ectopic pregnancy, pelvic pain and adhesion, menstrual abnormalities etc [27]. Demonstration of *Mycobacteria* on culture is used as gold standard and is the most specific test for the diagnosis of TB. But extra-pulmonary TB is pauci bacillary in nature and its practical utility is very limited due to very low sensitivity [28], [29]^.^ Therefore, it is mandatory to combine available tests to enhance its sensitivity and specificity in diagnosing genital tuberculosis [30]^.^ In the present study, a total of 78 endometrial tissue and/or aspirate were collected from suspected FGTB patients and detected as tuberculosis positive patients by Lowen-stein Jensen culture and or Z-N staining methods. Considering the case definition of *M. tuberculosis* positive cases used in this study, only 9 (11.5%) cases were diagnosed as positive TB cases. These findings correlated with Abebe *et al* (2004), who had reported 12% *M. tuberculosis* positivity among 25 suspected FGTB patients in Ethiopia. In this study only Z-N stain positive cases were found in 15.38 % cases [31]. Previously, Gupta (2016) reported of 8% AFB positive cases among suspected FGTB patients [32]. This high percentage of positivity of Acid fast bacilli (AFB) in Z-N stain could be explained by the fact that presence of any pathogenic or saprophytic mycobacteria of vaginal flora and skin in the sample [33]. Among the nine TB positive cases, 5.1% cases were only L-J culture positive and 6.4% cases were both L-J culture and Z-N staining positive. Closely similar finding were reported by Rozarti *et al* (2006) who had reported 7.8% culture positive cases [34]. Causes of low positivity in culture of endometrial tissue and/or aspirate might be the fact that pauci bacillary nature of extra pulmonary samples, uneven distribution of tubercle bacilli in the specimen and substantial number of the bacilli in the specimen might be bacteriologically dormant [35]. In this study, 12.5% infertility cases and 13.7% cases of ectopic pregnancy patients were diagnosed as genital TB. Djuwantono *et al* (2017) reported the prevalence of GTB in infertile population in developing countries to be between 5 and 20%. Together with endometrial involvement, TB of the fallopian tube is the leading cause of infertility in GTB [36]. Parasad *et al* (2012) reported 14.6% infertile women were diagnosed as genital tuberculosis [37]. High rate of Genital TB among infertility patients could be explained due to MTB infestation and endometrial receptivity could be impaired for blastocyst implantation [38].

Sharma *et al* (2014) reported 18 (13.2%) ectopic pregnancy cases concomitant with genital tuberculosis which is similar to the present study [15]. Banerjee *et al* (2012) reported 35.29% ectopic pregnancy as a cause of GTB (6 out of 17) in Northern India [16]. In genital tuberculosis, fallopian tubes and endometrium are consistently involved. Tortuous fallopian tube and reduced uterine receptivity is very common. Occlusion in isthmus and ampulla region of fallopian tube and loss of cilliary function may play an important role in ectopic pregnancy [38], [39].

In this study, highest percentage (77.77%) of TB cases had no sign symptom of tuberculosis and similarly Kour *et al* (2018) described among 50 genital tuberculosis patients 66% (33/50) were asymptomatic [17]. This proves that in the early stage of disease patients might be diagnosed as asymptomatic. Majority (56.4%) of the patients in the present study resided in the urban areas. The possible reasons are that women of the urban society are now much cognizant regarding ectopic pregnancy and infertility management [40], [41].

FGTB occurs mostly in reproductive age and after puberty the blood supply to the pelvic organs is increased. More bacilli can reach and infect the reproductive organs [34]. In the present study, the age range of the patients was 22 to 40 years and most of the cases (41%) were found below thirty years. Thangappah *et al* (2011) and Hatami (2005) found most of the patients (83.3% and more than 30 %) of genital tuberculosis in their study less than thirty years of old [12], [42].

Endometrial receptivity is altered by the presence of mycobacterium and cause of implantation failure through mechanisms such as disturbed immunomodulation and cytokine overburden, endocrine disruption, activation of anti-phospholipid antibodies, and micro thrombosis without the presence of overt clinical disease [30].

In the present study, two pro inflammatory cytokine TNFα and IFNγ were analyzed. Both the cytokines were significantly higher among TB positive patients (p-value = 0.01) than the TB negative patients. It may indicate towards hostility of endometrium, possibly leading to implantation failure. Datta *et al* (2018) reported significantly high value of IFN- γ in endometrial tissue and/or aspirate among TB positive patients. There is an established relationship between IFN- γ and tuberculosis. MTB infestation causes increased level of IFNγ which may be a helpful indicator of local presence of MTB [26]. Essone *et al* (2014) reported higher frequency of IFN-γ secreting T cells among TB positive cases than TB negative group [43]. Among study participants, 15.38% of patients were considered as presumptive for TB cases but there were no significant relationship between IFNγ and TNFα expression among them.

In the present study, ROC curve was analyzed for IFN- γ and TNF-α expression among TB positive and TB negative patients. The cut-off value for TNFα and IFN- γ detected from the curve was 10 and 23.5 respectively. The diagnostic sensitivity, specificity of TNF-α was 66.7%, 73.1% and IFN- γ sensitivity, specificity was 66.7% and 89.3%. The area under curve (AUC) value for TNFα was 0.767 and IFN- γ was 0.757. This study was consistent with other study finding, where area under curve (AUC) value for TNFα and IFN- γ were 0.641 and 0.694 respectively.

Any form of MTB bacillus can change the endometrial environment and may cause reproductive failure by cytokine release. The present study level of IFN γ and TNFα were significantly higher in diagnosed genital tuberculosis patients and had higher sensitivity and specificity.

## 5 Conclusions

IFN-γ and TNF-α were significantly higher among tuberculosis positive patients. But due to a small sample size in this study it would be unwise to make a conclusive remark increased expression of studies cytokines among TB positive patients. More studies, possibly with more samples, would be necessary to establish a positive correlation between TB positivity and increased expression of endometrial cytokines.

**Data availability statement:**

All data used in this study are available upon reasonable request from corresponding author

**Ethical statement:**

The objectives of the study, procedure, risks, benefits, and privacy issues were explained to the patients. A written informed consent was taken from the participants. All data were masked and stored in a password protected laptop. The study was ethically approved by Institutional Review Board at BSMMU.

## Declaration of Competing Interest

The authors declare that they have no known competing financial interests or personal relationships that could have appeared to influence the work reported in this paper.
